# Leptin Receptor Deficiency Protects Mice against Chronic Cerebral Hypoperfusion-Induced Neuroinflammation and White Matter Lesions

**DOI:** 10.1155/2020/7974537

**Published:** 2020-12-18

**Authors:** Yu Du, Yufei Song, Xiaojie Zhang, Yan Luo, Wenying Zou, Jiawei Zhang, Jianliang Fu

**Affiliations:** Department of Neurology, Shanghai Jiao Tong University Affiliated Sixth People's Hospital, 600 Yishan Road, Shanghai 200233, China

## Abstract

Leptin participates in the inflammatory responses in multiple cell types and animal models. Chronic cerebral hypoperfusion (CCH) induces inflammation in the central nervous system (CNS), which acts as one of the main reasons for CCH-induced white matter lesions (WMLs). But whether leptin participates in the pathogenesis of CCH-induced WMLs remains unknown. Therefore, we performed bilateral common carotid artery stenosis (BCAS) to induce CCH on the leptin receptor- (LepR-) deficient db/db mice, aiming to evaluate the possible involvement of leptin in CCH-induced cognitive impairment, WMLs, and neuroinflammation, and further explore the effect of leptin on chronic hypoxia-induced inflammation using the BV2 microglial cell line. After four weeks of BCAS, wild-type mice showed significant working memory deficits, WMLs, activation of microglia and astrocytes, decrease in the number of oligodendrocytes, downregulation of myelin basic protein expression, and increase in the expression of TNF-*α* and IL-1*β*; however, four weeks of BCAS failed to induce significant changes in the LepR-deficient db/db mice but elevated the production of anti-inflammatory cytokines and activated the M2 microglia. We further confirmed that leptin would aggravate the hypoxia-induced proinflammatory cytokine expression in the BV2 microglia cell line. These results suggested that LepR deficiency would protect mice against the CCH-induced cognitive impairment and WMLs by inhibiting glial activation and suppressing proinflammatory responses as well as promoting anti-inflammatory cytokine expression and M2 microglia activation in the white matter.

## 1. Introduction

White matter hyperintensities (WMHs) are commonly observed in the brain scanning of the elderly population, which reflect the pathology of white matter lesions (WMLs) [1]. WMLs occur due to chronic cerebral hypoperfusion (CCH) and the subsequent chronic cerebral hypoxia. It could contribute to cognitive decline and even dementia [2]. However, the mechanisms and modulating factors involved in the CCH-induced WHLs are still not fully understood.

CCH induces chronic inflammation in the central nervous system (CNS), and inflammation might be the mediator of CCH-induced WMLs [3]. Microglia are the immune cells in CNS. They act as the first line of defense against pathogens, clearing up cellular debris, expressing inflammatory cytokines [4]. It was demonstrated that suppressing inflammatory responses and inactivating microglia ameliorated CCH-induced cognitive dysfunction and WMLs [5].

Leptin is predominantly produced by adipose tissue. It functions through the leptin receptor (LepR) and was originally recognized as the modulator of food intake and metabolism. However, growing evidence suggested that leptin also participated in the inflammatory responses and immunity, regulating the production of several cytokines [6]. Leptin also plays an important role in CNS, targeting multiple cell types via LepR [7]. It was confirmed that microglia could express LepR and produce proinflammatory cytokines under leptin stimulation [8]. But whether leptin participates in the pathogenesis of CCH-induced WMLs remains unknown.

In this study, we performed bilateral common carotid artery stenosis (BCAS) to induce CCH in the LepR-deficient db/db mice, aiming to evaluate the possible involvement of leptin in CCH-induced cognitive impairment, WMLs, and neuroinflammation, and further explore the effect of leptin on chronic hypoxia-induced inflammation using the BV2 microglial cell line.

## 2. Materials and Methods

### 2.1. Animals

Male db/db mice (BKS.Cg-Dock7m +/+Leprdb/Nju) and wild-type mice (C57BLKS/JNju) were purchased from Nanjing Biomedical Research Institute of Nanjing University (Nanjing, China). Mice were housed in cages with a 12 h light/dark cycle and were given access to food and water ad libitum. All the surgical and behavioral procedures were conducted according to the National Institutes of Health for the care and use of laboratory animals in scientific investigations and approved by the Ethics of Animal Use in Research Committee of Shanghai Jiaotong University School of medicine. Db/db mice have been proved to carry the homozygous spontaneous mutations in the LepR and are resistant to leptin [9]. There were differences in the body weight between WT and db/db mice. The bodyweight of the wild-type mice we used in the experiment was 21.7 ± 3.2 g, and the bodyweight of db/db mice was 58.6 ± 5.1 g.

### 2.2. Animal Models of Chronic Cerebral Hypoperfusion

Chronic cerebral hypoperfusion (CCH) was induced by bilateral common carotid artery stenosis (BCAS) using microcoils as described elsewhere [10]. These microcoils were made of piano wire with an inner diameter of 0.18 mm, a pitch of 0.50 mm, and a total length of 2.5 mm (Sawane Spring Co., Shizuoka, Japan). For the BCAS surgery, mice aging 12-16 weeks were anesthetized with 2% isoflurane and then maintained with 1.5% isoflurane in 70% N_2_ and 30% O_2_, with a heating pad to maintain the rectal temperature within 37 ± 0.5°C. Through a midline cervical incision, both common carotid arteries (CCAs) were exposed and freed from the vagal nerves. Two 4-0 silk sutures were placed around the distal and proximal parts of each CCA to make a lift. Then, microcoils were rotated around the bilateral CCAs just below the carotid bifurcation. Mice in the sham group simply went through these procedures without applying the microcoils.

### 2.3. Experimental Groups of Animal Models

Db/db mice and wild-type mice were randomly assigned into four groups as follows: (1) sham-operated wild-type mice (wild-type sham); (2) BCAS-operated wild-type mice (wild-type BCAS); (3) sham-operated db/db mice (db/db sham); (4) BCAS-operated db/db mice (db/db BCAS). Mice from the BCAS-operated groups were euthanized at three time points for different experiments. After three days and one week of BCAS, mice were directly euthanized for the subsequent analysis. After four weeks of BCAS, mice were executed following the behavioral test.

### 2.4. Cell Culture and Treatment

The BV2 microglial cell line was purchased from ScienCell (CA, USA) and cultured in MEM medium with 10% FBS and 1% penicillin/streptomycin. For normoxia culture, cells were maintained in a humidified incubator containing 95% air and 5% CO_2_ at 37°C; for hypoxia culture, cells were maintained in a tri-gas incubator (Thermo Fisher Scientific, USA) with oxygen concentration at 1%. BV2 cells were cultured in the normoxia/hypoxia condition with/without the treatment of mouse recombinant leptin (Peprotech, UK) at the concentration of 1 *μ*M for 24 h.

### 2.5. Measurement of the Cerebral Blood Flow (CBF)

Under deep anesthesia of isoflurane described above, mice were placed in a ventral recumbent position on a heating pad, and the rectal temperature was maintained between 36.5-37.5°C. The scalp and the periosteum were removed to expose the skull, and then a thin layer of glycerin was applied on the skull to keep it moisturized. Next, the CBF was monitored through the skull with a laser speckle blood flow imager (Pericam, Sweden) for four min. The region of interest was a 3 × 5 mm^2^ rectangle located between the bregma and the lambda, and the measuring height of the laser probe was 10 cm. CBF of mice from different groups was repeatedly recorded at different time points.

### 2.6. Behavioral Test for Spatial Working Memory

Y-maze test was conducted in the four groups of mice at the end of week four to evaluate spatial working memory. Briefly, each mouse was placed in the center of a Y-shape maze, which consists of three equal arms (30 cm long × 10 cm high × 8 cm wide) and was allowed to explore freely within 8 min. Each arm that the mouse entered was recorded, and the exploration of three arms successively was a correct alternation. The percentage of spontaneous alternations was calculated as the number of correct alternations divided by the total number of alternations [the number of correct alternations/(the total number of arms entries‐2) × 100%].

### 2.7. Preparation for Histological Experiments

After four weeks of BCAS or sham operation, wild-type mice and db/db mice were anesthetized with pentobarbital sodium and transcardiac perfused with precooled PBS and 4% PFA (in PBS). The whole brain was harvested and postfixed in 4% PFA at 4°C for 6 h and subsequently dehydrated in 20% and 30% sucrose. After that, the mouse brain was embedded in optimal cutting temperature (OCT) (Sakura, USA) and sliced into consecutive coronal sections (thickness of 10 *μ*m or 20 *μ*m per section) from bregma +1.10 mm to bregma -0.22 mm at -20°C in a cryostat (Leica, Germany). These sections were equally divided into three groups according to their location: the anterior, middle, and posterior part of the corpus callosum. Every time one section from each part with the same intervals in each mouse was chosen for the histological experiments.

### 2.8. Measurement of WMLs

The Klüver–Barrera (KB) staining was used to measure the degree of WMLs. We used 10 *μ*m sections with the same intervals from each mouse as mentioned above, and the KB staining was performed as previously described [11]. The severity of white matter lesions was determined as normal (grade 0), disarrangement of the nerve fibers (grade 1), formation of marked vacuoles (grade 2), or disappearance of myelinated fibers (grade 3) in the corpus callosum [12].

### 2.9. Immunofluorescence Staining

For immunofluorescence staining, 20 *μ*m sections were washed with 0.01 M phosphate-buffered saline (PBS) for 3 × 5 min and blocked in 5% normal donkey serum (Jackson, USA) with 0.3% Triton X-100 (Sigma, USA) for 30 min at room temperature. The sections were then incubated with goat anti-Glial Fibrillary Acidic Protein (GFAP) antibody (Abcam, UK, 1 : 500), goat anti-ionized calcium-binding adaptor molecule-1 (Iba-1) antibody (Abcam, UK, 1 : 500), rabbit anti-Olig2 antibody (Proteintech, China, 1 : 100), rabbit anti-MBP antibody (Abcam, UK, 1 : 1000), rat anti-CD16/32 (BD Bioscience, USA, 1 : 200), or rabbit anti-CD206 (Abcam, UK, 1 : 1000) overnight at 4°C. After washing with PBS for 3 × 5 min, the sections were then incubated with immunofluorescent secondary antibodies (Abcam, UK, 1 : 500) for 30 min in darkness at room temperature. The sections were then washed with PBS for 3 × 10 min and incubated with DAPI (CST, USA) for 5 min. Next, the sections were mounted with mounting media (Vector Labs, USA) and imaged with a fluorescence microscope (Olympus, Japan) as soon as possible. Cell numbers, the areas of the corpus callosum, and the mean OD intensities were counted and calculated with ImageJ software.

### 2.10. Real-Time PCR

Wild-type mice and db/db mice with BCAS operation for 3/7/28 days and sham operation were euthanized and transcardiac perfused with precooled PBS solution. The whole brain was then harvested, and the total corpus callosum was dissected under a stereomicroscope on ice. The cerebral cortex was removed as much as possible. The total RNA of the corpus callosum and cultured BV2 cells was extracted using Trizol reagents (Beyotime, China) and reverse-transcripted using the ReverTra Ace qPCR RT Master Mix with gDNA Remover kit according to the manufacturer's protocol (Toyobo, Japan). The real-time PCR was conducted using the SYBR Green Real-time PCR Master Mix kit (Toyobo, Japan) and the ABI 7500 FAST real-time PCR system (Applied Biosystems, USA). The primers are as follows (5′-3′): tumor necrosis factor-*α* (TNF-*α*), forward-CCACCACGCTCTTCTGTCTAC, reverse-AGGGTCTGGGCCATAGAACT; interleukin-10 (IL-10), forward-GCTGCCTGCTCTTACTGACT, reverse-CTGGGAAGTGGGTGCAGTTA; interleukin-1*β* (IL-1*β*), forward-TGCAGCTGGAGAGTGTGGATCCC, reverse-TGTGCTCTGCTTGTGAGGTGCTG; interleukin-6 (IL-6), forward-CTGCAAGAGACTTCCATCCAG, reverse-AGTGGTATAGACAGGTCTGTTGG; interleukin-4 (IL-4), forward-AACGAGGTCACAGGAGAAGG, reverse-CTGTGGTGTTCTTCGTTGCT; interleukin-1 receptor antagonist (IL-1Ra), forward-GTGAGACGTTGGAAGGCAGT, reverse-GCATCTTGCAGGGTCTTTTC; GAPDH, forward-GAACGGGAAGCTCACTGG, reverse-GCCTGCTTCACCACCTTCT.GGAGCTGTCATTAGGGACATCA.

### 2.11. ELISA

Cell culture supernatants were harvested for ELISA (Multisciences, China) according to the manufacturer's instruction to detect the secreted TNF-*α* and IL-1*β* protein levels by BV2 cells under normoxia and hypoxia conditions with or without the presence of leptin.

### 2.12. Statistical Analysis

Statistical analysis was performed using IBM SPSS Statistics 22.0, and graphs were plotted using GraphPad Prism 6.0 software. Data were expressed as the mean ± SEM. The CBF changes were compared using one-way ANOVA with repeated measurements following Fisher's LSD post hoc test; data from the behavioral test, histological examination, immunofluorescent staining, real-time PCR of cell samples, and ELISA were analyzed using one-way ANOVA following Fisher's LSD post hoc test. The statistical analysis methods of inflammatory cytokines real-time PCR data of animal models were explained in the legend.

## 3. Results

### 3.1. CBF Changes

**Figure 1:**
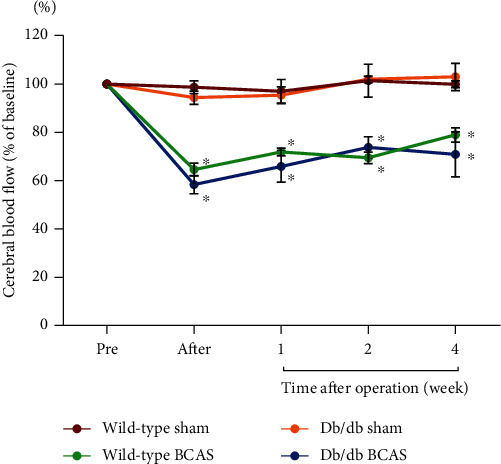
Cerebral blood flow (CBF) changes in different groups of mice. CBF was measured at the following time points: preoperation, right after the operation, one, two, and four weeks after the operation. CBF value was expressed as the percentage of baseline CBF value for each animal. Data were presented as the mean ± SEM. Pre: pre-BCAS/sham operation; after: right after BCAS/sham operation. *N* = 5, ^∗^*p* < 0.05 compared with sham.

To verify that CCH was successfully induced and to examine the magnitude of the blood flow drop-off in mice from different groups, the CBF was measured at five time points: pre-BCAS/sham operation, right after the operation, one week, two weeks, and four weeks after the operation. The CBF values at different time points were expressed as the percentage of the baseline mean perfusion values. It was shown that the CBF values dropped greatly after BCAS in both wild-type and db/db groups and recovered overtime gradually but were still lower than preoperation (Figure 1). Compared with sham groups, there was a significant CBF decline in the BCAS groups both in wild-type and db/db mice at any time points after BCAS, while the CBF values failed to change after the sham operation (Figure 1).

### 3.2. Spatial Working Memory after BCAS

**Figure 2:**
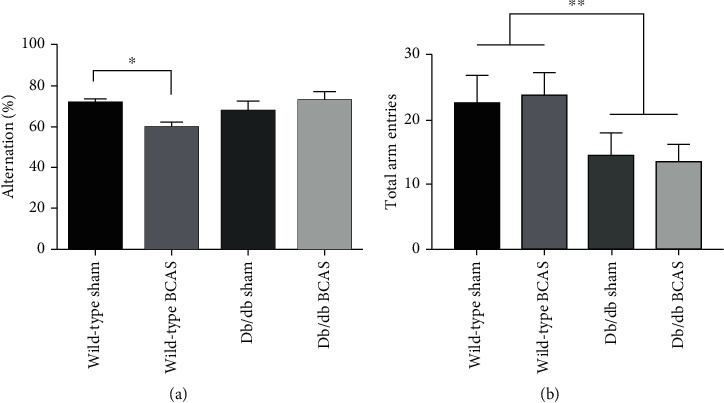
Spatial working memory in mice from different groups. (a) Histogram represented the alternation outcome of mice that underwent the 8-minute Y-maze test. (b) The number of total arm entries of mice from different groups in the Y-maze test. *N* = 9 − 14, data were presented as the mean ± SEM. ^∗^*p* < 0.05, and ^∗^*p* < 0.01.

Given the same scale of CBF decline among wild-type mice and db/db mice, it was then explored whether the CBF decline would lead to a certain cognitive deficit. After four weeks of BCAS, we performed a Y-maze test to evaluate the spatial working memory in mice among different groups. Impaired spatial working memory was observed in wild-type mice after four weeks of BCAS, as demonstrated by a reduction in the percentage of alternation behavior. Interestingly, compared with sham-operated db/db mice, there were no further cognitive deficits in db/db mice with BCAS (Figure 2(a)). The number of total arm entries within 8 min of db/db mice was lower than wild-type mice, and there was no difference between BCAS and sham operation (Figure 2(b)). These results suggested that db/db mice that carried the dysfunctional LepR mutation and were resistant to leptin might be protected from the CCH induced cognitive impairment.

### 3.3. Histological Changes in White Matter

**Figure 3:**
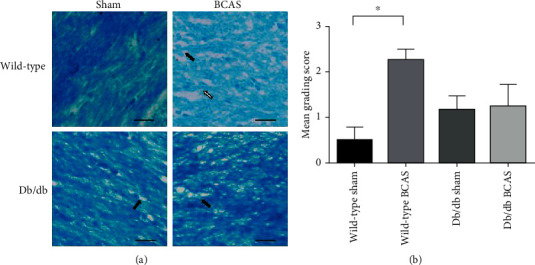
White matter lesions (WMLs) in wild-type and db/db mice with sham or BCAS operation for four weeks. (a) WMLs were evaluated using Klüver–Barrera (KB) staining. White matter vacuoles were shown by the black arrows, and the disappearance of myelinated fibers was shown by the white arrow. (b) Histogram showing the mean grading scores of WMLs. *N* = 4 − 5, scale bar = 20 *μ*m, and ^∗^*p* < 0.05.

WMLs in wild-type and db/db mice with the sham operation or four-week BCAS were determined by KB staining. Four weeks of BCAS induced tremendous lesions in the corpus callosum of wild-type mice, such as disarrangement of nerve fibers, formation of white matter vacuoles, and even the disappearance of myelinated fibers (Figure 3). There was a slight formation of white matter vacuoles in sham-operated db/db mice. However, comparing with sham-operated db/db mice, db/db mice that underwent four weeks of BCAS did not show any further WMLs (Figure 3).

### 3.4. Changes of Glial Cells in White Matter

**Figure 4:**
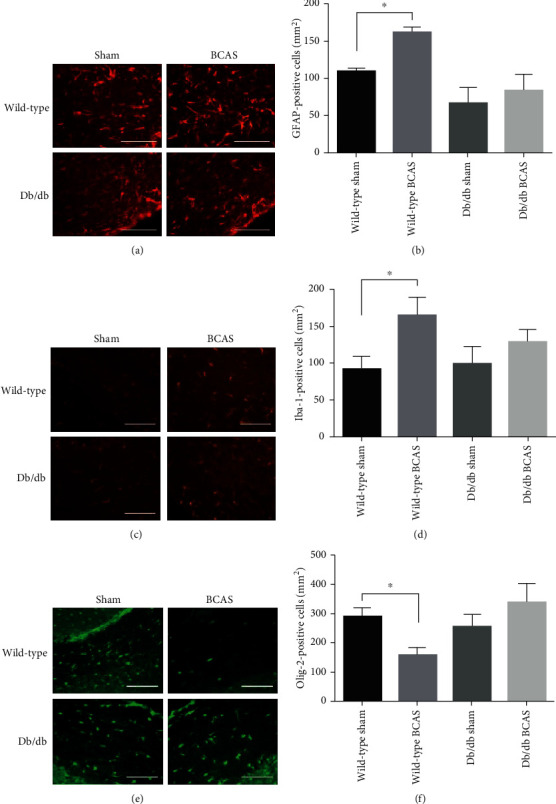
Immunofluorescence staining of glial cells in the corpus callosum of wild-type and db/db mice with sham or BCAS operation for four weeks. (a) Representative images of GFAP-positive astrocytes. (b) Histogram of GFAP-positive astrocytes density in different groups. (c) Representative images of Iba-1-positive microglia. (d) Histogram of Iba-1-positive microglia density in different groups. (e) Representative images of Olig-2-positive oligodendrocytes. (f) Histogram of Olig-2-positive oligodendrocytes density in different groups. *N* = 3 − 5, scale bar = 100 *μ*m, and ^∗^*p* < 0.05.

Since there were not significant WMLs in db/db mice after BCAS as in wild-type mice, we further explored the glial activation in mice from different groups. The number of Iba-1-positive microglia (Figures 4(a) and 4(b)) significantly increased in wild-type mice after BCAS. In contrast, the amount of microglia in sham-operated db/db mice and db/db mice after BCAS revealed no difference. The same trend was observed in the number of GFAP-positive astrocytes (Figures 4(c) and 4(d)). As is known, oligodendrocytes form the sheath of myelin fibers and play an important role in the normal functioning of white matter. The changes of Olig-2-positive oligodendrocytes were then examined (Figures 4(e) and 4(f)) by immunofluorescence staining. Oligodendrocytes decreased dramatically after BCAS in wild-type mice, associated with poor cognitive performance in the Y-maze test. But there was no evident shrinkage of the oligodendrocyte population in db/db mice after BCAS. These results corresponded to the behavior and histological tests, indicating that LepR deficiency might protect mice from the CCH-induced significant WMLs and cognitive deficits as occurred in wild-type mice through inactivating glial cells in the white matter.

### 3.5. Changes in MBP Expression

**Figure 5:**
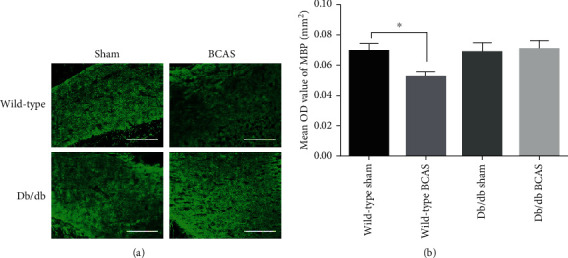
MBP expression in the corpus callosum of wild-type and db/db mice with sham or BCAS operation for four weeks. (a). Immunofluorescence staining of MBP. (b). Histogram showed the mean OD value of MBP in mice from different groups. *N* = 3 − 5, scale bar = 100 *μ*m, and ^∗^*p* < 0.05.

To further verify the different responses of wild-type mice and db/db mice to BCAS in terms of demyelination, the expression of MBP was examined by immunofluorescence staining. Consistent with the changes in oligodendrocytes, it was shown that the MBP level severely decreased in wild-type mice after four weeks of BCAS. However, there was still no significant change in BCAS-operated db/db mice than sham (Figure 5).

### 3.6. Changes in the Expression of Proinflammatory and Anti-Inflammatory Cytokines and M1/M2 Phenotype in Corpus Callosum

**Figure 6:**
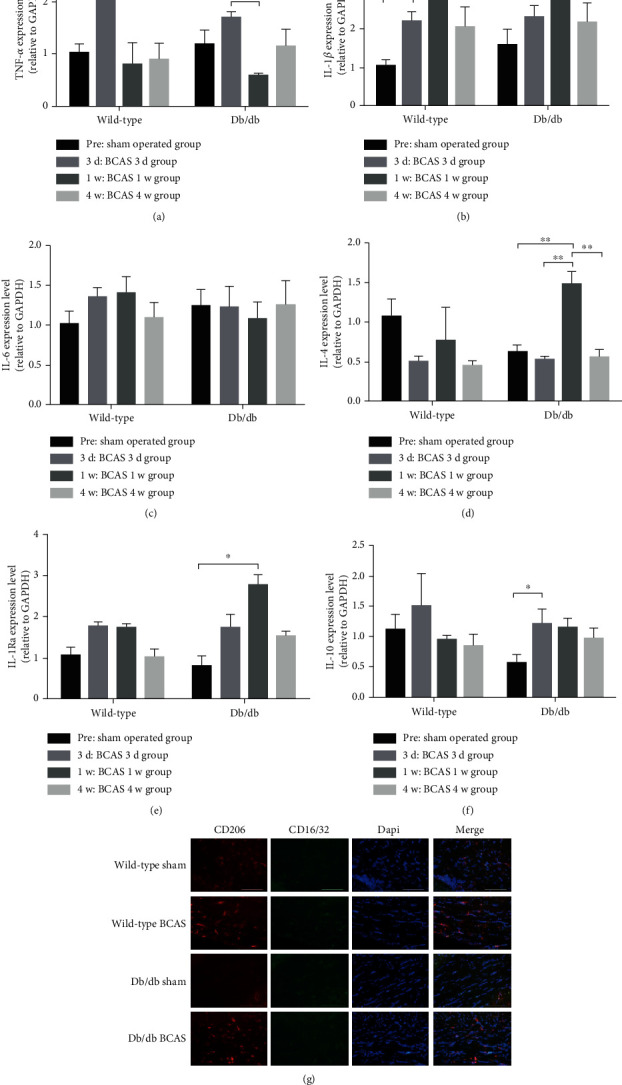
Expression of inflammatory cytokines in the corpus callosum of wild-type and db/db mice with sham or BCAS operation for four weeks. Relative mRNA expression levels of proinflammatory cytokines TNF-*α* (a), IL-1*β* (b), and IL-6 (c), and anti-inflammatory cytokines IL-4 (d), IL1Ra (e), and IL-10 (f) against GAPDH were examined by real-time PCR. M1/M2 microglial phenotypes in the mice corpus callosum were examined by immunofluorescence staining. Anti-CD16/32 antibodies were used to label M1-positive microglia, and anti-CD206 antibodies were used to label M2-positive microglia (g). In (a–d), comparisons between different time points were analyzed using Fisher's LSD test; in (e), comparison between different time points was analyzed using Dunnett's test; in (f), comparison between different time points in the wild-type group was analyzed using Kruskal Wallis test, and comparison between different time points in db/db group was analyzed using Fisher's LSD test. *N* = 3 − 5, scale bar = 100 *μ*m, ^∗^*p* < 0.05, and ^∗∗^*p* < 0.01. Wild-type: wild-type mice, Db/db: db/db mice.

A previous study indicated that leptin would facilitate proinflammatory cytokine production in microglia [8]. We speculated that lacking LepR might modulate the cytokine expression profile in db/db mice after BCAS and regulate the cognitive behavior and histological performance. The corpus callosum of wild-type mice and db/db mice with the sham operation and BCAS operation for 3/7/28 days was dissected, and the expression of several kinds of representative proinflammatory cytokines (TNF-*α*, IL-1*β*, and IL-6) and anti-inflammatory cytokines (IL-10, IL-4, and IL-1Ra) was examined using real-time PCR. The expression of TNF-*α* in wild-type mice increased significantly after three days of BCAS, then it decreased to the same level as the sham group, while TNF-*α* expression level did not elevate after BCAS in db/db mice at any time point (Figure 6(a)). IL-1*β* was elevated at all time points after BCAS in wild-type mice, but it did not increase in db/db mice after BCAS as much as the wild-type, with the only increase after seven days of BCAS (Figure 6(b)). The expression levels of IL-6 in different groups were unaltered (Figure 6(c)). As for the anti-inflammatory cytokines (Figures 6(d)–6(f)), the expression levels of IL-10, IL-4, and IL-1Ra did not change in the wild-type mice after BCAS at any time points, whereas in the db/db mice, all of them exhibited upregulation at certain time points. These results indicated that LepR deficiency might protect mice from CCH-induced brain damage through suppressing proinflammatory cytokine expression and promoting anti-inflammatory cytokine expression. Then, the microglial phenotypes were analyzed by immunofluorescence staining. It was found that the amount of both CD16/32-positive M1 microglia and CD206-positive M2 microglia in the corpus callosum was increased in wild-type mice after BCAS operation; however, in db/db mice, the amount of CD206-positive M2 microglia significantly increased after BCAS operation, but CD16/32-positive M1 microglia were inhibited (Figure 6(g)).

### 3.7. Leptin Promoted Proinflammatory Cytokine Expression in BV2 Cells under Hypoxia Condition

**Figure 7:**
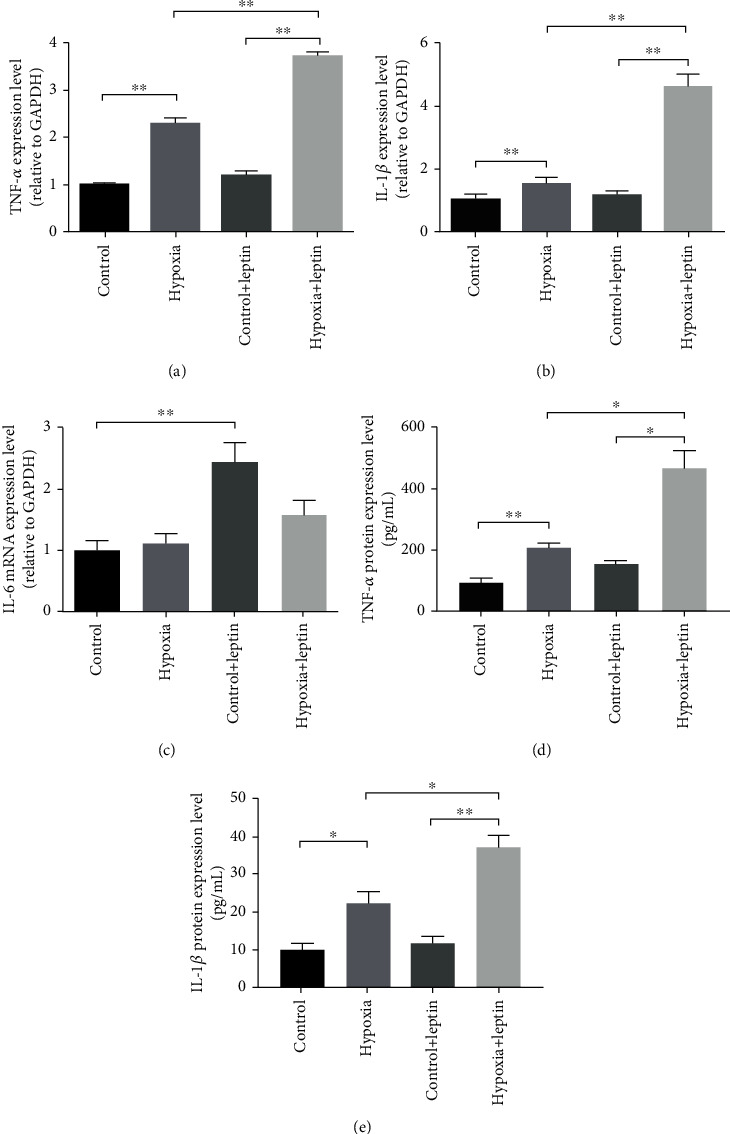
Expression of proinflammatory cytokines TNF-*α* (a) and IL-1*β* (b) in BV2 cells under normoxia/hypoxia conditions with/without leptin treatment for 24 hours. The mRNA expression levels of TNF-*α* (a), IL-1*β* (B), and IL-6 (c) were examined by real-time PCR, and the protein expression levels of TNF-*α* (d) and IL-1*β* (e) were examined by ELISA. *N* = 3, *p* < 0.05, and ^∗∗^*p* < 0.01.

As we have shown that deficiency of LepR suppressed the CCH induced proinflammatory cytokines expression, we then wanted to confirm whether leptin would promote the proinflammatory cytokine expression in microglia under hypoxia condition in return. We cultured BV2 cells under normoxia/hypoxia (1% oxygen concentration) conditions with/without leptin (1 *μ*M) treatment for 24 h and harvested cells for real-time PCR and ELISA examination. We found that hypoxia stimulated TNF-*α* and IL-1*β* mRNA and protein expression in BV2 cells, and leptin treatment further promoted the expression of TNF-*α* and IL-1*β*; however, hypoxia failed to modify the expression of IL-6 in BV2 cells, although leptin treatment triggered the expression of IL-6 (Figure 7).

## 4. Discussion

Previous studies indicated that leptin would facilitate proinflammatory cytokine production in microglia and other cell types as well as animal models [8, 13]. Microglia are the major source of cytokine production in the CNS, and CCH-induced neuroinflammation is believed to be an important pathogenic mechanism of CCH-induced WMLs and cognitive impairment. It brought us to think about the potential role of leptin in the CCH-induced WMLs. In this study, we first provide evidence that leptin signaling plays a pivotal role in the pathogenesis of CCH-induced WMLs. We found that LepR deficiency would protect mice from the CCH-induced cognitive impairment and WMLs by inhibiting glial activation and suppressing proinflammatory cytokine expression as well as promoting anti-inflammatory cytokine expression in the white matter. And we further confirmed that leptin would aggravate the hypoxia-induced proinflammatory cytokine expression in the BV2 microglia cell line.

We successfully induced mild CCH by performing BCAS for four weeks in wild-type mice and db/db mice, revealed by the CBF reduction. We chose the Y-maze test and the 8-minute alternation protocol to test the spatial working memory of mice from different groups. BCAS would lead to poor Y-maze 8-minute alternation performance [14, 15], which was further verified in our study. Although many studies have addressed the neuronal and cognitive-behavioral features of db/db mice, Sharma et al. suggested that there was no spatial working memory deficit in db/db mice [16]. In our study, we confirmed that db/db mice had no spatial working memory deficit compared with wild-type mice and found that db/db mice after four weeks of BCAS showed no further spatial working memory impairment. Db/db mice presented several characteristics of metabolic syndrome, such as overweight and high blood glucose. But we did not observe connections between high blood glucose and chronic cerebral hypoperfusion-induced spatial working memory impairment. So, we did not emphasize the overweight of db/db mice. Concerning that BCAS cerebral hypoperfusion model mainly affects the integrity and function of the corpus callosum and leads to spatial working memory dysfunction [17], we chose Y-maze 8-minute alternation to evaluate the cognitive performance in different groups as previously reported [18]. The results indicated that mild CCH decline could not induce a cognitive decline in the LepR-deficient db/db mice as it did in the wild-type mice, and LepR deficiency might protect the db/db mice from the severe damage that CCH would have caused. We noted that the number of total arm entries of db/db mice was lower than wild-type mice. We speculated that this was because of the lower motor ability of db/db mice due to overweight.

The activation of microglia and astrocytes and the decline of oligodendrocyte density and MBP expression are the common features of CCH-induced WMLs [19]. Activated microglia would participate in the white matter damage through releasing proinflammatory cytokines such as TNF-*α* and IL-1*β* [5]. However, the lack of LepR might lead to poor microglia and astrocyte responsiveness against disturbances such as trauma, hypoxia, and inflammation [20, 21]. In our study, we found that LepR dysfunction protected the mice from the CCH-induced histological damages in the white matter, ameliorating the CCH-stimulated expression of proinflammatory cytokines such as TNF-*α* and IL-1*β*, and further upregulated the expression of anti-inflammatory cytokines such as IL-4, IL-10, and IL-1Ra. As microglia can be simplistically divided into the proinflammatory M1 microglia and the anti-inflammatory M2 microglia [22], the microglial phenotypes were then examined using the phenotype markers: CD16/32 (M1) and CD206 (M2). Consistent with the cytokine expression profile, the anti-inflammatory M2 microglia in db/db mice dominated after the BCAS operation. Furthermore, cell experiments revealed that leptin would further upregulate the hypoxia-induced proinflammatory cytokine expression. These results manifested that lack of LepR, which would mediate the proinflammatory signaling pathway in the CNS, prevented the proinflammatory microglia's participation in the pathogenesis of white matter damage. Nevertheless, the mechanisms through which LepR stimulation and hypoxia work synergistically to induce TNF-*α* and IL-1*β* production in BV2 cells remain to be further elucidated. It was reported that in Raw 264.7 cells, phospholipase D1-JNK pathway would mediate the leptin-induced TNF-*α* expression [23].

There are certain limitations in our study that we will address in our future experiments. We failed to provide evidence of whether the leptin receptor knockdown in the BV2 cells would mimic the specific changes of cytokine production observed in this study in vivo. Moreover, the outcomes of this study are all observational, and the molecular mechanisms of leptin modulating cytokine production remain to be elucidated.

## Data Availability

The data used to support the findings of this study are available from the corresponding author upon request.
